# Two Bonebridge bone conduction hearing implant generations: audiological benefit and quality of hearing in children

**DOI:** 10.1007/s00405-021-07068-x

**Published:** 2021-09-08

**Authors:** Soňa Šikolová, Milan Urík, Dagmar Hošnová, Vít Kruntorád, Michal Bartoš, Oldřich Motyka, Petr Jabandžiev

**Affiliations:** 1 Department of Pediatric Otorhinolaryngology, University Hospital Brno, Černopolní 9, 61300 Brno, Czech Republic; 2 Faculty of Medicine, Masaryk University Brno, Kamenice 5, 62500 Brno, Czech Republic; 3 Nanotechnology Centre, CEET, VSB-Technical University of Ostrava, 708 00 Ostrava-Poruba, Czech Republic; 4 Centre ENET, CEET, VSB-Technical University of Ostrava, 708 00 Ostrava-Poruba, Czech Republic; 5 Department of Pediatrics, University Hospital Brno, 61300 Brno, Czech Republic

**Keywords:** Active transcutaneous bone conduction implant, Children, Quality of life, Atresia, Localisation, Hearing outcomes

## Abstract

**Purpose:**

The study aimed to evaluate audiological benefits, quality of hearing and safety of two Bonebridge generation: BCI601 and BCI602 (MED-EL, Innsbruck, Austria) in children.

**Methods:**

Twelve children were implanted: five BCI601 and seven BCI602 comprising of ten conductive hearing loss, and two single sided deaf SSD subjects. Audiological outcomes tested were sound field audiometry, functional gain, speech recognition threshold (SRT50), speech recognition in noise (SPRINT) and localisation abilities. Subjective measures were Speech, Spatial and Qualities of Hearing Scale (SSQ12).

**Results:**

The mean FG with the BCI601 was 25.0 dB and with the BCI602 28.0 dB. The benefit in SRT50 was 23.2 dB and 33.8 dB, respectively. The mean benefit in SPRINT was 15% and 6.7% and the localisation ability improved from 33.3° to 16° and from 26.2° to 17.6°, respectively. The two SSD subjects reported a FG of 17 dB, a benefit in SRT50 of 22.5 and a benefit in SPRINT of 20%. Subjective outcomes improved significantly and even exceeded the values of their age-and sex matched normal hearing peers. One revision was reported: a retroauricular emphysema above the implant occurred 12 months post-OP, it was resolved operatively with the implant still being functional.

**Conclusion:**

The pediatric cohort reports significant audiological benefit, even exceeding that of the age- and sex matched control. The combination of the high safety and audiological benefit makes the Bonebridge a comfortable and effective option in hearing rehabilitation in children.

## Introduction

In general, all implant recipients exhibit a wide range of speech perception skills with a range of factors identified affecting clinical performance despite of the degree and type of hearing loss [1]. Hensch et al. showed that the capacity for plasticity in the response properties of neurons in- and consequently, the functional organization of cortical and sub-cortical sensory structures was maximal within ‘critical periods’ during early development [2]. Not surprising then, the significantly better auditory level of performance between pre-lingually compared to post-lingually or even pre-lingually late CI-treated children [3–6]. The importance of recovering hearing loss in the pediatric population as fast as possible was investigated widely for several different hearing implants and results showed that ongoing hearing loss leads to deficits in psychomotor development (cognitive, emotional, motor, and social capacities). Hence, early treatment of hearing loss is not only important for auditory performance but also necessary for the social and educational development, which is accompanied by high patient satisfaction and improved quality of life. Bone conduction implants have particularly benefited people with mild to moderate conductive and combined hearing loss (C/MHL). The first active transcutaneous bone conduction implant, the Bonebridge (BCI601, MED-EL, Innsbruck, Austria), launched in 2012, was up till 2019, when Cochlear launched their OSIA system, the only active system which is placed with the skin intact. It is composed of an external audio processor and a bone conduction floating mass transducer (BC-FMT) placed transcutaneously into the temporal bone. The BCI601 is a CE and FDA approved option for children aged 5 years and older to restore CHL and Single-Sided-Deafness (SSD), with bone conduction thresholds at 45 dB HL or better [7]. The first-generation BCI601 has been investigated in numerous studies which have been systematically reviewed by Magele et al., showing the significant and stable benefit of the device as well as the long-term safety, expecially when compared to its percutaneous competition devices [8]. The transcutaneous technology of the BCI601 avoids the typical high complication rates involved in percutaneous bone conduction devices [8, 9]. Furthermore, technically, the active system of the BCI601 still poses the most advanced option in treating CHL, since it combines the benefit of direct stimulation (same audiological output as percutaneous systems) with the benefit of reduced skin complications of transcutaneous systems [10]. Compared to BAHA and Ponto percutaneous implants, the two main disadvantages of the BCI601 are the size of the implant that reduce the indications in young children and the artefact produced by the implant [11]. Even though the size of the BC-FMT in the new generation, the BCI602, was reduced, requiring a drilling depth compareable to that of a BAHA-screw, still in patients with comorbid intracranial tumour or cholesteatoma necessitating regular imaging control with MRI the artefact may is a disadvantage compared to percutaneous implants. Recent studies by Edlinger et al. and Utrilla et al. adressed this possible problem by investigating artefact reduction possibilities with the BCI602 and concluded, that with the application of artefact reduction sequences and certain anatomical placements also tumour- and cholesteatoma cases can be diagnosed succesfully [11, 12]. Especially, the reduced depth of the implant makes pre-surgical planning redundant and with the new MRI possibilities open new possibilities for difficult anatomies as well as the option to implant children younger than 5 years of age [13, 14].

The aim of this study was to evaluate the audiological outcomes, benefits, and safety of the two generations, the BCI601 and BCI602, implanted in twelve children (five and seven, respectively). To the best of knowledge of the authors, this is the second study on the new Bonebridge BCI602 [15], as well as the first comparison to its precursor generation.

## Materials and methods

### Study population

**Fig. 1:**
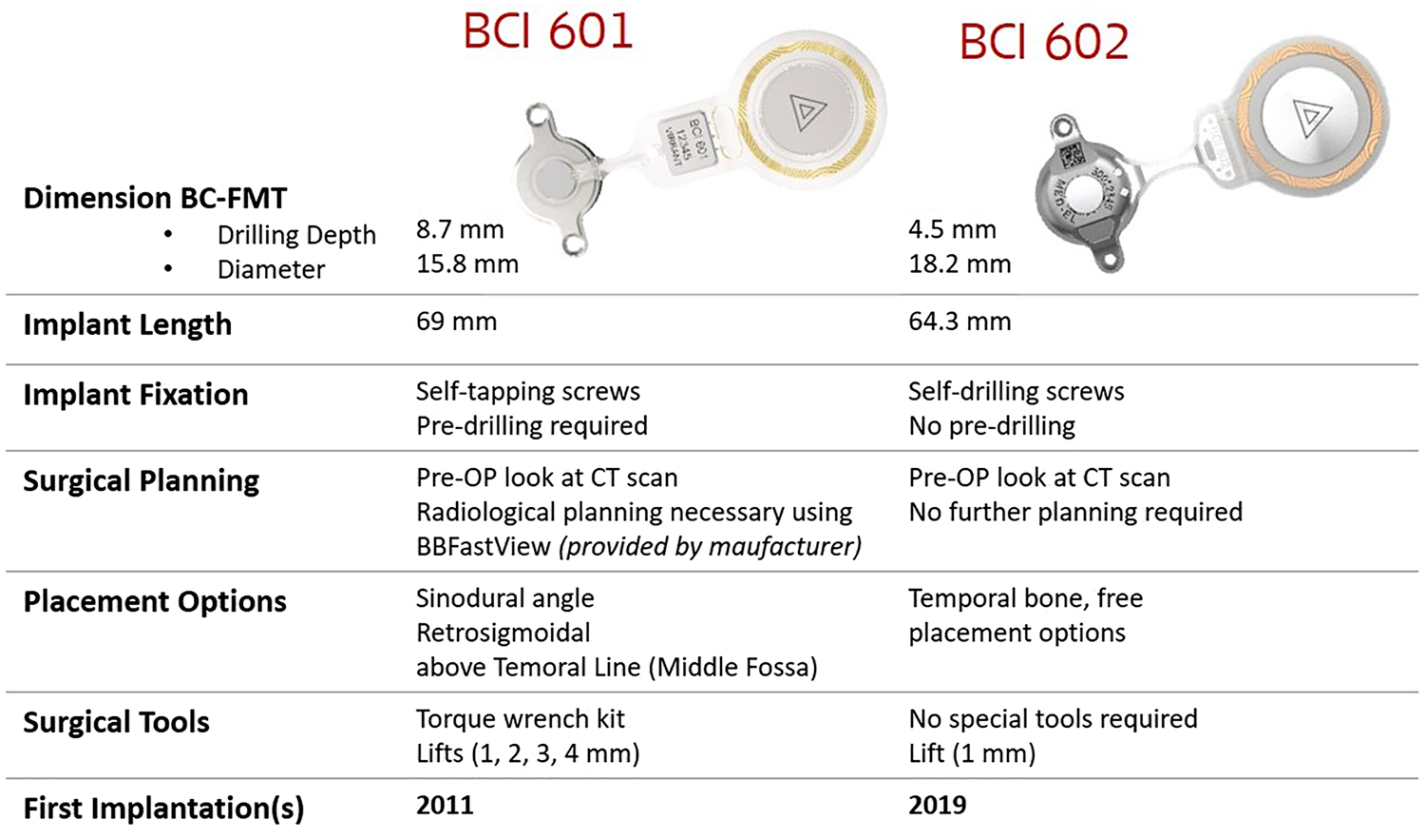
Overview of the main differences of the BCI devices (*left*) colum shows the BCI 601 and (*right*) colum depicits the BCI602 characteristics which are different (unmentioned characteristics are identical). (*implant pictures courtesy of MED-EL*)

The prospective data analysis and implantation was performed as part of routine clinical procedures between January 2018 and December 2020 at the tertiary centre. The study protocol was approved by the ethics committee of University Hospital (No. 03-041,120) and informed consent of the parents/legal guardian was given prior surgical intervention. The audiological inclusion criteria were based on manufacturer’s recommendations and paediatric patients suffering from CHL and SSD were included (Fig. 1).

### Audiological evaluations

All audiometric tests were performed pre-operative (pre-OP) and 3 months post-operative (post-OP) in a soundproof audiometric booth, using the audiometer Interacoustics AC40E (Denmark, 2019).

Pure tone measurements were performed at a frequency range from 0.5 to 4 kHz. Pure tone average air (PTA4AC) and bone (PTA4BC) conduction hearing thresholds were calculated as the mean of the evaluated AC and BC values at 0.5, 1, 2 and 4 kHz.

**Fig. 2:**
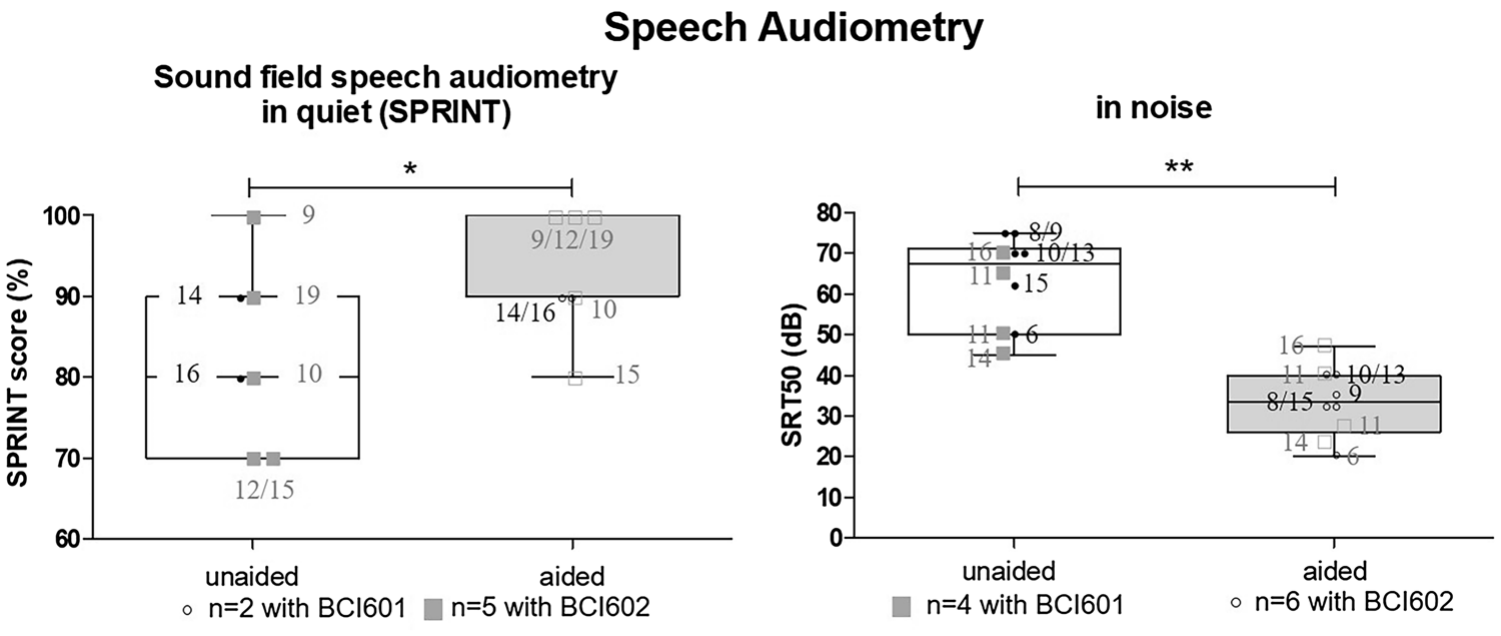
Speech Audiometry outcomes presented in box-plots showing outcomes for the total cohort (*left*) speech reception measurements in noise SPRINT (%) (*right*) results for measurements in quiet using SRT50 (dB). White boxes represent the pre-operative, and the grey box the aided condition: mean, median and SD. Circles show individual values of BCI601 and squares individual outcomes of BCI602 users. Pre-OP values are depicited in full and post-OP values in open symbols. Numbers next to the respective individuls represent the age at implantation

Sound-field thresholds were measured using frequency—modulate warble tones presented from the aided side, with the loudspeaker positioned 3 m away from the subject. Soundfield audiometry (SF), speech recognition threshold (SRT50) and speech recognition in noise test (SPRINT) at 65 dB HL in a multi-talker babble were performed. The contralateral ear was masked with narrowband noise during pure-tone and sound field audiometry, and with broad band noise during the speech tests. The noise level was determined by the experienced audiologist as necessary. All audiological examinations were performed with (aided) and without the bone conduction hearing device (unaided) and with temporary hearing aid used before implantation (previous HA). In four children with CHL, localisation testing was performed with a circular, ten loudspeaker arrangement. The localisation test tool v.1.0 provided by MED-EL was utilized, using a stimulus of different white noise types and a stimulus level of 40 dB. The outcomes were separated into the stimulus provided from the front or from the back for better visualisation purposes displayed in Fig. 2.

### Hearing-related questionnaire

**Fig. 3:**
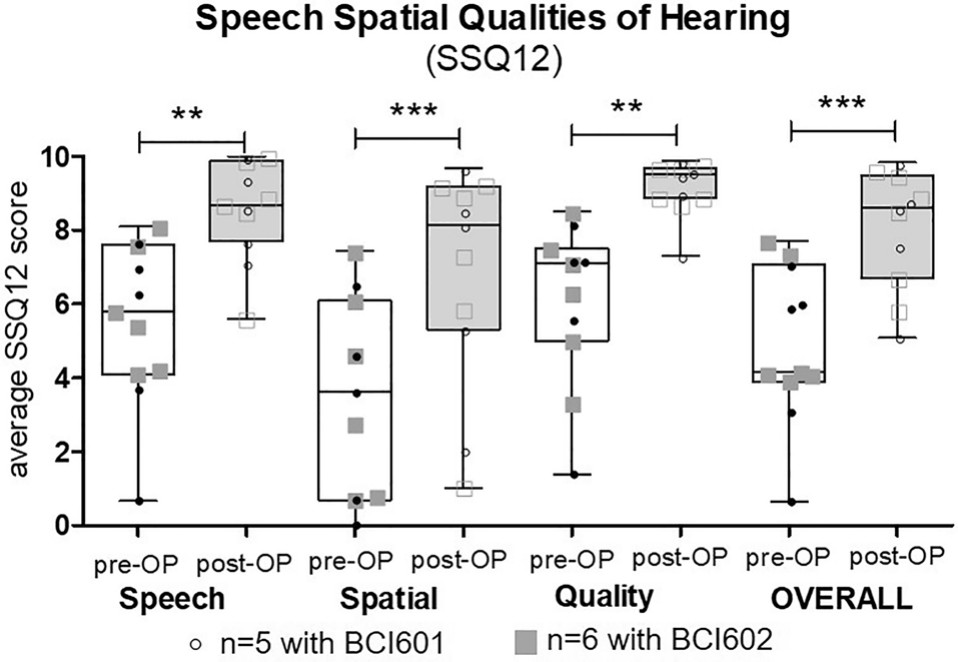
Speech, Spatial Qualities of Hearing (SSQ12). Subscores of speech, spatial hearing and perceptive qualities as well as the total score comparing pre-OP to post-OP results. Horizontal lines denote the median. There was a highly statistically significant improvement in each subscore. Circles show individual values of BCI601 and squares individual outcomes of BCI602 users. Pre-OP values are depicited in full and post-OP values in open symbols

The Speech, Spatial and Qualities of Hearing Scale (SSQ 12) questionnaire was designed to measure auditory disability across a wide variety of domains, reflecting the reality of hearing in everyday life [16]. Items are scored on a visual analogue scale from of 1–10, with higher numbers representing greater satisfaction. Apart from a total score, the SSQ 12 provides three subscores for speech, understanding, spatial hearing and other qualities of hearing [16]. The questionnaire was completed by the child with his parents to assess their hearing ability before and after implantation (pre- vs post-OP) (Fig. 3).

### Data analysis

**Fig.4:**
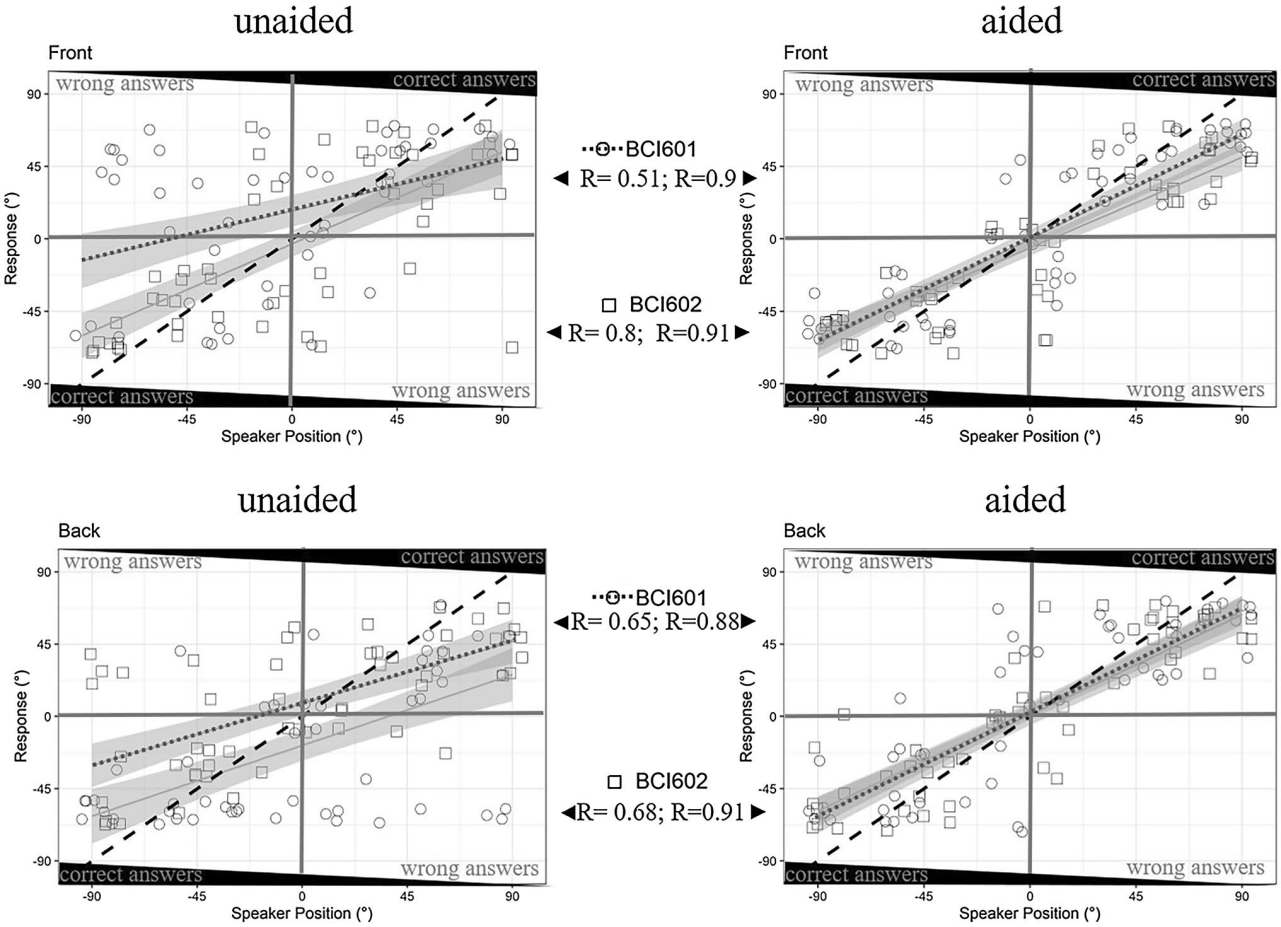
The localisation ability. The accuracy of sound localisation in the horizontal plane in the unaided (*left*) and aided condition (*right*) [front (*top*) vs. back (*bottom*) sound source] is displayed. The speaker position is given in degrees (°) on the *x*-axis and is plotted against the answers of the users on the *y*-axis. Black dotted line indicates normal-hearing outcomes and Pearson *R* gives the correlation effectiveness, respectively of the device generation used. Circles show individual values of BCI601and squares individual outcomes of BCI602 users

Descriptive analysis was used to report demographics (e.g. age and gender), baseline characteristics (e.g. aetiology), and patient-reported outcomes mean, SD, median, minimum and maximum (Table 1). The non-parametrically distributed outcomes were analyzed using GraphPad Prism 7.0 statistical software. The Wilcoxon signed-rank test to evaluate significant differences between unaided (pre-OP), previous HA aided, and Bonebridge aided (post-OP) pure-tone, sound-field, SRT50 and SPRINT outcomes was applied. Scores from the SSQ12 were analysed using the one-sample Wilcoxon signed-rank test to test for significant difference. Speech audiometry (Fig. 2) as well as Questionnaire outcome (Fig. 3) are displayed in Box-plots with the ends of the box representing the upper and lower quartiles (interquartile range), the vertical line inside the box marks the median and the whiskers extend from the highest to the lowest observation. The individual outcomes are displayed as circles or square within the box-plot. The localisation outcomes were analysed using R Statistical Computing Environment using the metafor package [17]. Stimulus response plots were generated for the unaided vs the aided condition and the results of the stimulus response relationship was quantified by the line of best fit. The correct answers with sound stimulus from the front were separated from the sounds coming from the back and a Pearson’s *R* score was generated to test for the correlation efficiency. The Root Mean Square Localisation Error (RMSE) was calculated as a function of unaided versus aided conditions, for both implant generations separated as well as for signals presented from the back and from the front (°) (Table 3; Fig. 4).

**Table 1:** Patient demographics

ID	Age (y)	Sex	Side	PTA Ipsilateral (dB HL)		PTA Contralateral (dB HL)		Etiology	Previous HA
				AC	BC	AC	BC		
P1	11	M	R	59	10	60	9	AA BILAT	BAHA Soft/ ADHEAR
P2	14	F	L	58	10	20	8	AA LEFT	ADHEAR
P3	16	F	R	59	10	18	10	AA RIGHT	ADHEAR
P4	11	F	L	60	20	45	20	AA BILAT	BAHA Softband
P5	12	F	R	110	80	10	0	SSD RIGHT	Cross. ADHEAR
P6	6	F	L	58	11	46	10	AA RIGHT	BAHA Softband
P7	19	M	L	101	85	21	15	SSD LEFT	Cross. ADHEAR
P8	9	M	L	54	8	20	5	AA LEFT	ADHEAR
P9	10	M	R	68	8	13	8	AA RIGHT	ADHEAR
P10	13	M	R	69	16	86	27	CWD BILAT	Cross
P11	8	F	L	64	14	64	16	AA BILAT	BAHA Softband
P12	15	M	L	60	8	16	9	AA LEFT	Contact mini

PTA denotes the pure-tone average threshold for 0.5. 1. 2. and 4 kHz in dB HL

*AA *aural atresia, *AC *air conduction, *BC *bone conduction, *CWD* canal wall down, *F *female, *L *left, *M *male, *R* right, *HA* hearing aid

## Results

**Fig.5:**
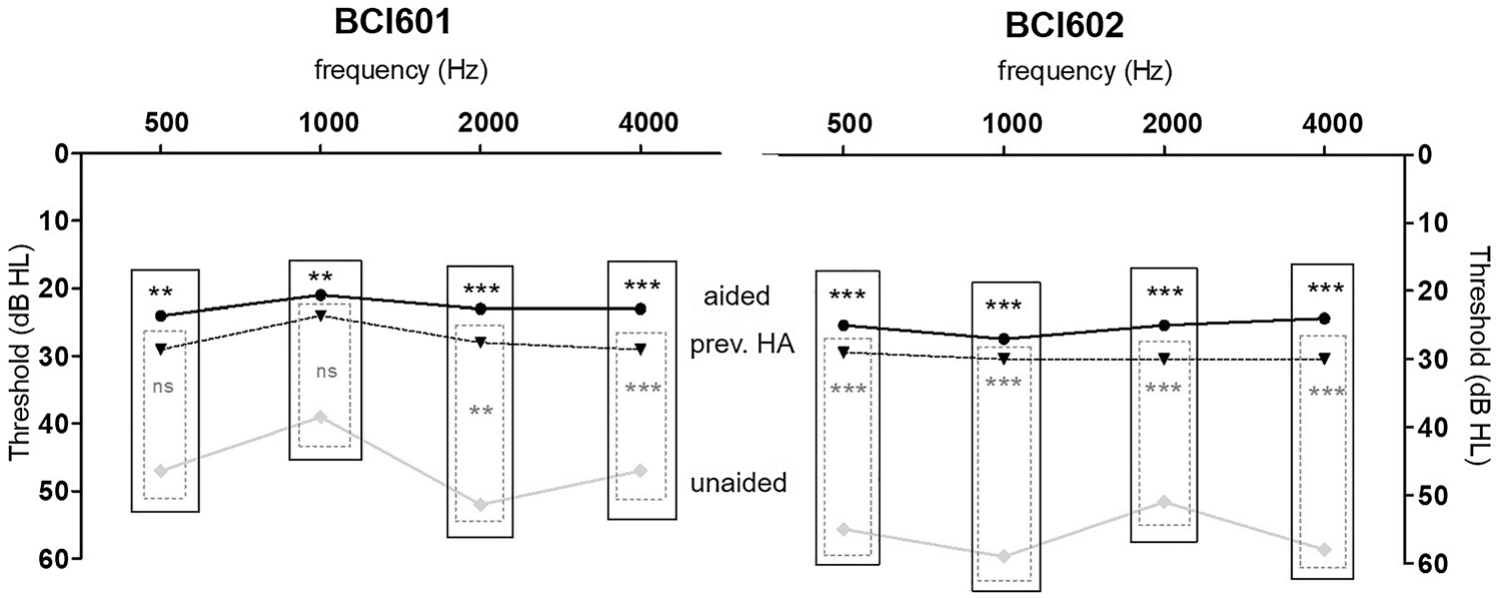
The BCI601 and BCI602 Soundfield measures for the BCI601 (*left*) and for the BCI602 (*right*). Symbols and lines indicate the mean pre-op AC, previous Hearing Aid (HA) and post-operative Bonebridge aided situation, respectively

In total, ten paediatric patients suffering from CHL [with the majority of the children suffering from aural atresia (*n* = 9)] and two from SSD were included. Five were implanted with the BCI601 and seven with the BCI602 (MED-EL, Innsbruck, Austria). The two SSD subjects have each received a BCI601 and a BCI602. The study cohort comprised of six females and six males. The mean age at implantation was 12 ± 3.5 years, ranging from the youngest with 6 years up to 19 years of age. Prior to surgery all subjects trialled different Bone Conduction Hearing Devices, such as the ADHEAR, BAHA Softband, Cross Hearing and Contact Mini. Even though Soundfield outcomes with the previous HA were quite satisfying (Fig. 5), most of the users either opted for an implantable solution due to cosmetic issues, or because speech understanding was, especially in challenging situations (classroom, sports, etc.), difficult. The study population received unilateral implantation, even though four suffered from bilateral hearing loss. At the time of last follow-up, the average experience with the device was 15.6 ± 8.2 months, with a maximum of 30 months (BCI601) and a minimum of 6 months after implantation. Detailed demographical information is summarised in Table 1, and Table 2 describes surgical and post-OP complication details.

**Table 2:** Preoperative course and device fitting using models BCI601 and BCI602

ID	Age (years)	Device	Pre-OP planning	Position of the FMT	Type of screw	Use of lift	Exp. dura mater	Comp. sig. sinus	Post-op complications not procedure related	Revision
P1	11	BCI601	Yes	Transmastoid	Standard	–	–	–	–	–
P2	14	BCI601	Yes	Transmastoid	Emergency	✓	–	–	–	–
P3	16	BCI601	Yes	Transmastoid	Standard	–	–	✓	–	1 year post-op: retroauricular emphysema (insertion of fat and fibrin glue around the BC-FMT)
P4	11	BCI601	Yes	Transmastoid	Emergency	–	✓	–	–	–
P5	12	BCI601	Yes	Transmastoid	Standard	–	–	–	–	–
P6	6	BCI602	No	Transmastoid	Standard	–	–	–	–	–
P7	19	BCI602	No	Transmastoid	Standard	–	–	–	4th day post-op: acute OM (*antibiotics*)	–
P8	9	BCI602	No	Transmastoid	Emergency	–	–	–	1st day post-op: torticollis (*proc. by Bemmer*)	–
P9	10	BCI602	No	Transmastoid	Standard	–	✓	–	–	–
P10	13	BCI602	No	Transmastoid	Standard	–	–	–	–	–
P11	8	BCI602	No	Transmastoid	Emergency	–	–	–	1st day post-op: cough (*Stoptussin gtt*)	–
P12	15	BCI602	No	Transmastoid	Standard	–	–	–	–	–

All post-op complications were resolved; in parenthesis () the resepective treatment, *Exp*. exposure of, *Comp*. *sig*. compression of sigmoid sinus, *OM* Otitis Media

### Surgical outcomes and complications

For the BCI601, optimal placement of the BC-FMT was planned via pre-OP CT scans using the 3-d planning software BBFastView of the temporal bone (kindly provided of MED-EL). In all patients, the transmastoidal (TM) approach was carried out. For the BCI602 placement, only pre-OP CT scans were visually inspected without further planning. No complications occurred during surgery. BCI lift was used only in one patient implanted with a BCI601. In four subjects, the emergency screw had to be used because of an inferiorly localised screw due to a highly pneumatised mastoid tip and thin superficial bone. In two patients, the dura was exposed (BCI601 and BCI602) and compressed by 1 mm. The sigmoid sinus was compressed in one patient (BCI601). Detailed peri-operative course and device models BCI601 or BCI602 are depicted in Table 2. Only one patient with Eustachian tube dysfunction and significantly lower weight (BMI—Body Mass Index—16) experienced a local complication one year after surgery. The female adolescent (16 years of age at implantation) developed a retroauricular emphysema above the implant, communicating through the auditus ad antrum and mastoid to the subcutaneous tissue. This late complication was solved by suction of air bubbles from the pocket and by sealing the artificial opening around BC-FMT with fat from the earlobe and fibrin glue. Since the revision, no further air has accumulated in the retroauricular area and the implant has been fully functional [18]. No patient-reported pain or irritation of the skin at or around the implant side.

### Objective-audiology results

The outcomes of the two SSD subjects reported pre-OP mean in SRT50 of 52.5 ± 17.1 which improved to 30 ± 14.1 after Bonebridge treatment (one device generation each). The mean SPRINT outcomes exhibited pre-operative mean level of 80 ± 14.14% and improved to a mean of 100% after implantation. The pre-OP SF was 39 ± 1.8 dB HL and with previous HA 26 ± 0.88 dB HL and improved to 22 ± 0.88 dB HL. Due to the low number and the equally distributed outcomes, the SSD subjects were not separately according to their device analysed nor was statistical analysis possible. The analysis of the CHL cohort implanted with the BCI601 resulted in a significant improvement from the mean pre-OP value of hearing threshold in sound field of 48.00 ± 6.6 compared to the BB-aided condition 23.00 ± 3.7 dB HL (*P* = 0.098), but was not significantly different when compared to previous hearing aids used 28.0 ± 2.4 (*P* = 0.125) (Table 3). The analysis for the BCI601 implanted subjects resulted in a significant improvement from the mean pre-OP value of hearing threshold in sound field (SF) of 53.00 ± 12.0 compared to the aided condition with 25.00 ± 4.1 dB HL (*P* = 0.036) and was significantly different when compared to previous hearing aids used 29.0 ± 5.5 (*P* = 0.036) (Table 3). Differences were also seen across the frequencies, especially the benefit of amplification at 0.5 and 1 kHz was more beneficial in the BCI602 cohort compared to the BCI 601 (Fig. 5). The average functional gain with the BCI601 was of 25.0 ± 5.15 dB and with the BCI602 the results exhibited 28.0 ± 8.05 dB. The BCI601 aided mean value measured in four individuals of SRT50 improved from 57.5 ± 11.9 dB in the unaided condition to 34.3 ± 11.2 dB in the aided condition (not significant, *P* = 0.125). For the six subject with the BCI 602, the mean SRT50 improved from 67.0 ± 9.6 dB in the unaided condition to 33.2 ± 7.4 dB in the aided condition (*P* = 0.031). Merging the ten subjects resulted in a highly significant improvement in SRT50 (*P* = 0.006).

**Table 3:**
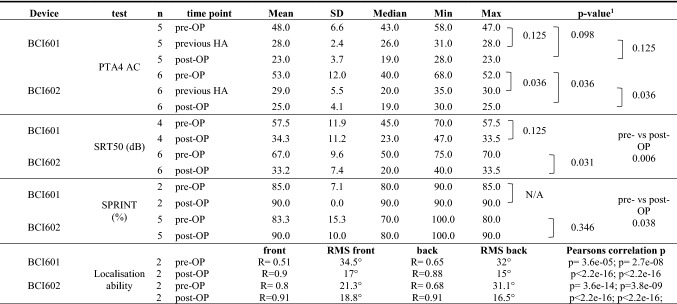
Objective: soundfield/speech audiometr

*SF* sound field, *SD* standard deviation, *SRT* speech recognition threshold, *SPRINT* speech recognition in noise test, *HA *hearing aid; pre- vs post-OP compares the total population

^1^*t* test, Wilcoxon signed-rank test

The speech Speech Recognition in Noise Test (SPRINT) exhibited in the BCI601 cohort (*n* = 2) a mean of 85.0 ± 7.1% and improved to 90.0 ± 0.0% post-OP (statistics N/A). In the BCI602 study group, five subjects understood with the device 90% ± 10.0%, which improved from 83.3 ± 15.3 in the pre-OP condition. Only the total CHL cohort comprising of seven measured subjects showed a statistically significant improvement with a *P* value of 0.038.

Tests of sound-source localization were conducted in four subjects, two implanted with the BCI601 and two with the BCI602. Localization was tested in the aided and unaided condition and outcomes separated into sound presented from the front versus sound presented from the back. Figure 4 displays the speaker position given in degrees (**°)** on the *x*-axis is plotted against the answers of the users, shown as the stimulus response plots. The correct answers with sound stimulus from the front barely correlated with the trend line resulting in a Pearson *R* of 0.51 for the BCI601 and with the BCI602 a Pearson *R* of 0.8 in the unaided condition was observed. These outcomes improved in the BCI601 aided condition to *R* = 0.8 and *R* = 0.91. Sound localisation ability with the stimulus given from the back resulted in a correlation of the trend line in the BCI602 and BCI602 of *R* = 0.65 and *R* = 0.68 in the unaided conditon and improved to *R* = 0.88 and *R* = 0.91 in the aided conditon, respectively. The calculated root mean square error (RMSE) with signals from the front in the unaided condition was 34.5° and improved to 17° in the aided condtion for the BCI 601. Similar results were observed in the BCI602 implanted subjects with signals from the front (18.8° and 21.3°). Investigating the results for the BCI601 with signals presented from the back a RMSE of 32° was observed in the unaided condition which improved to 15° in the aided condition. Similar results were seen for the BCI602 subjects, were the RMSE improved from 31.1° when unaided to 16.5° in the aided situation (Table 3).

### Subjective-questionnaire results

The overall SSQ12 score for the total cohort revealed a highly significant subjective benefit (*P* = 0.0005) which was also seen for the subcategories of Speech, Spatial and Qualities of Hearing (*P* = 0.0019; *P* = 0.0005; *P* = 0.0019, respectively). The rating for the BCI601 implanted cohort was also significantly better when compared to the pre-OP condition (*P* = 0.063), which was rated even better in the BCI602 implanted study group (*P* = 0.031). The sames device-respective statistical significance was found in the subdomains of Speech, Spatial and Qualities of Hearing measured (Tables 3, 4).

**Table 4:**
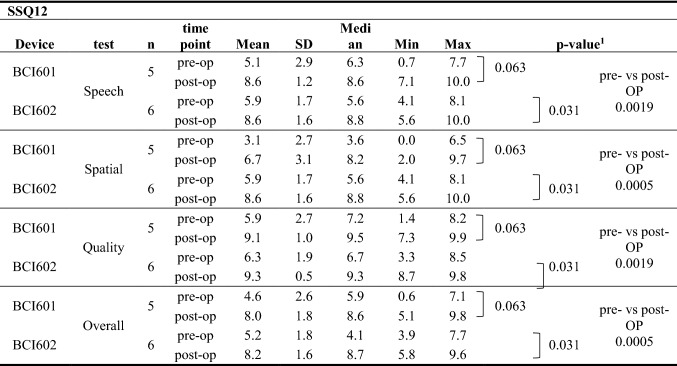
SUBJECTIVE: Questionnaire results

^1^ *t* test. Wilcoxon signed-rank test; pre- vs post-OP compares the total population

## Discussion

The primary goal of this study was to evaluate the audiological benefits, quality of hearing and safety in children implanted with the Bonebridge implant system. Since the first implantation of the BCI601 in 2018, the patients report beneficial audiological outcomes, hence high patient satisfaction, accompanied by low complications rates, very similar to those reported in the literature [8]. We were, therefore, obviously interested in investigating the new generation of the device, the BCI602, in our pediatric patient cohort as well as exploring possible differences between the two device generations.

Audiological performance tests were chosen to best reflect everyday hearing situations, hence real-life benefit. The beneficial rehabilitation of hearing should also be reflected in post-operative subjective evaluation of quality of hearing, which in in our experience was often lacking a correlation of objective measures, such as speech reception and/or localisation ability, and the patient’s objective expectations and self-assessment of their hearing abilities. We, therefore, utilized the Speech, Spatial and Qualities of Hearing via a short questionnaire (SSQ12). Pleasingly, our subjective audiological measures go hand in hand with the subjective, patients’ self-assessment: showing significantly improved localisation abilities paired with significantly improved dimension of spatial hearing in the questionnaire—outcomes apply for both device generations. On the other hand, we observed, that the pre-operative bone conduction hearing aid trial of up to three months was in terms of SF measurements quiet successful, nonetheless the patients still opted for an implantable solution. We conclude this from the fact (and after correspondence with the parents), that either the stigmatisation, especially in that particular age-group was too high (mean age at implantation was 12 ± 3.5 years), the speech understanding, particularly in challenging environments such as classrooms and at parties, was not sufficient enough and/or the wearing comfort was not given, as for most of those devices, except the ADHEAR, high pressure for optimal sound transmission through the skin is required [19]. A wide range of non-implantable devices were trialled, from the first pressure-free bone conduction hearing device, the ADHEAR to the well-known Softband versions of the BAHA, up to Mini Contact (Table 1). Even though the benefit with the trial-devices was significant compared to the unaided condition, the rehabilitation was not as significant and satisfactory in all tests applied, subjective as well as objective, compared to the post-operative outcomes for both bone conduction implant device generations. Outcomes reported in the literature form children reached an average aided sound field threshold close to normal hearing with the BCI601, i.e. 24 dB HL for 67 implants [20–22] which is similar to our observations (23.0 ± 3.7 dB HL and 25.0 ± 4.1 dB HL). The Speech perception in quiet (SRT50) resulted in significant benefit of 11% for both generations (*P* = 0.0382). The SRT50 significantly improved in the BCI602 group with an average gain of 33.8 dB after implantation. The lack of significant benefit in the BCI601 group, even though the outcomes improved to 34.3 dB (gain 23.2 dB) may be again due to the relatively low number of subjects (*n* = 4). Nonetheless, these outcomes are in accordance with the recent reported literature [8]. The patients with the lowest hearing benefit were not those suffering from SSD, but not surprisingly the ones implanted late (aged 14 and 16 at time of implantation), this is applicable for both device groups. Our results showed improvement of functional hearing after Bonebridge implantation in all twelve cases, independent of device generation and aetiology. Outcomes in children (subjects 18 years or younger) implanted with the BCI601 were reported only in a handful of publications [23]. Zernotti et al. investigated 14 congenital atresia patients implanted with the, at the time only available, active BCI601 and reported significant improvements in hearing thresholds and word recognition scores accompanied with low complication rates. Magele et al. reported from six studies in their meta-analysis on children with CHL or MHL an average FG of 34 dB [8, 24–26]. Especially, the sound localization ability, which was investigated with white noise presented at a level of 40 dB SPL from randomized angles of − 90°, − 45°, 0°, 45°, or 90° showed very pleasing results for both the testet cohort of CHL cases. Sound localization performance was quantified using the RMS error and revealed a benefit from the unaided to the aided conditon of almost 20° for both generations together. Surprisingly, Weiss et al. found no significant difference between the unaided and Bonebridge aided conditions for auditory localization in the horizontal plane in 18 subjects, which might be due to the seven loudspeaker set-up which might have been for the given task too close together [27]. Vyskocil et al. on the other hand found in five users that the Bonebridge improved sound localization significantly and that the benefit concerning sound-source localization was depended on the location of the sound source [28]. Our results showed no location of sound dependent outcome, at least not in the aided condition. Currently, subject numbers are too small to draw conclusions on the benefit of sound localization in the aided situation, but results clearly show less wrong answers in the aided condtion for both, the BCI601 as well as for the BCI602. This can be clearly seen in Fig. 4, where the respective quadrant of the left/right ear wrong answers is almost empty (upper left and lower right side). Very pleasing are the correlating results of the objective audiological measures when compared to the subjective impression of the young patients themselves. We analysed data from the SSQ12 questionnaire revealing improved hearing in all measured dimensions: speech, spatial, qualities of hearing, hence the overall—hearing-related QoL after Bonebridge implantation in children with CHL or SSD. Needless to say, that the advantages of the Bonebridge system are especially beneficial for children, in which the thickness and dimensions of the skull bone are not sufficiently strong. Additionally, in difficult anatomies, surgeons might be confronted with dura mater and/or sigmoid sinus exposure which may require to gain space for the BC-FMT by compressing the dura and/or sinus. The study by Vyskocil and colleagues systematically evaluated the audiological outcomes of patients the BCI601 directly coupled to the dura and/or sinus and concluded, that direct stimulation of the soft tissue structures under the skull provides satisfactory hearing outcomes without adverse events reported [29]. The first generation, BCI601, has already demonstrated lower incidence of skin complications in comparison with other BAHDs [8, 22, 30, 31] and it is expected to be similar or even better due to the reduced size in the new generation, the BCI602. The incidence of post-operative pain also has been reported as relatively low for the BCI601 [32], such complaints were not reported in our cohort, neither for the BCI601 nor for the BCI602. Among our patients, high satisfaction with the audiological benefit is communicated; comfort- and improved aesthetics of the Bonebridge with its low profile, especially in comparison to the pre-OP trial-devices, is reported, which is in coherent with the literature [33–40]. Most children also report very good results in communication and using the audio processor on a day-to-day basis, while parents especially appreciate the improvement in social interactions and speech development [30, 33, 34]. From a surgical perspective, the second-generation BCI602 was engineered from the ground up to deliver optimal surgical handling and reliable implant fixation**.** As compared to the previous model BCI601, the new BCI602 provides nearly 50% less drilling depth due to reduction of the BC-FMT thickness from 8.7 to 4.5 mm and flexible implant positioning, which opens up new possibilities for difficult pathologies up to implanting children younger than the age of five including a wide range of anatomies and underlying pathologies [7]. The main surgical approach in the literature is the transmastoid implantation, which is also the method of choice for our patient cohort. It proved to be easy, safe and proved satisfactory outcomes in our implanted subjects. In unfavourable anatomical conditions a retrosigmoid approach is chosen [41], the other option is middle fossa approach [8, 42]. So far, the only study from Canada in 2020 shows that there is no significant difference between the location of FMT transmastoidly or retrosigmoidally, as well as the use of different types of cortical fixation screws and lifts [42].

Most of the reports in the literature on the results of Bonebridge implantation in children have employed small study groups with a maximum of up to 20 patients. Our report uses a similar sample size, which is limitation of the study and further investigations with higher subject numbers should be employed in the future. Such reports might be brought together as part of a meta-analysis which could draw much stronger conclusions about the safety, efficacy and effectiveness of this solution for children.

## Conclusion

All children, as well as their parents, were very satisfied with both implanted generations of the Bonebridge: the BCI601 and the BCI602. Sound-field audiometry, speech audiometry, speech audiometry in noise as well as localisation abilities showed a significant benefit after implantation. Subjective assessment of hearing quality as investigated via the SSQ12 improved significantly after implantation. The combination of the high safety and significant objective as well as subjective benefit makes the Bonebridge (both generations) a comfortable and effective option for hearing rehabilitation in children suffering from CHL or SSD.

## Declarations

### Availability of data and materials

All data are available in the main author.

### Code availability

Not applicable for this section.

## References

[1] Blamey P, Arndt P, Bergeron F, Bredberg G, Brimacombe J, Facer G, Larky J, Lindstrom B, Nedzelski J, Peterson A, Shipp D, Staller S, Whitford L (1996). Factors affecting auditory performance of postlinguistically deaf adults using cochlear implants. Audiol Neurootol.

[2] Hensch TK (2004). Critical period regulation. Annu Rev Neurosci.

[3] Bruijnzeel H, Ziylan F, Stegeman I, Topsakal V, Grolman W (2016). A systematic review to define the speech and language benefit of early (< 12 Months) pediatric cochlear implantation. Audiol Neurootol.

[4] Colletti L, Mandala M, Colletti V (2012). Cochlear implants in children younger than 6 months. Otolaryngol Head Neck Surg.

[5] Dettman SJ, Dowell RC, Choo D, Arnott W, Abrahams Y, Davis A, Dornan D, Leigh J, Constantinescu G, Cowan R, Briggs RJ (2016). Long-term communication outcomes for children receiving cochlear implants younger than 12 months: a multicenter study. Otol Neurotol.

[6] Geers AE, Nicholas J, Tobey E, Davidson L (2016). Persistent language delay versus late language emergence in children with early cochlear implantation. J Speech Lang Hear Res.

[7] MedEl. https://www.medel.com/hearing-solutions/bonebridge, ed.

[8] Magele A, Schoerg P, Stanek B, Gradl B, Sprinzl GM (2019). Active transcutaneous bone conduction hearing implants: Systematic review and meta-analysis. PLoS ONE.

[9] den Besten CA, Monksfield P, Bosman A, Skarzynski PH, Green K, Runge C, Wigren S, Blechert JI, Flynn MC, Mylanus EAM, Hol MKS (2019). Audiological and clinical outcomes of a transcutaneous bone conduction hearing implant: Six-month results from a multicentre study. Clin Otolaryngol Off J ENT-UK Off J Netherlands Soc Oto Rhino Laryngol Cervico Fac Surg.

[10] Schwab B, Wimmer W, Severens JL, Caversaccio MD (2020). Adverse events associated with bone-conduction and middle-ear implants: a systematic review. Eur Arch Otorhinolaryngol.

[11] Utrilla C, Gavilan J, Garcia-Raya P, Calvino M, Lassaletta L (2021). MRI after Bonebridge implantation: a comparison of two implant generations. Eur Arch Otorhinolaryngol.

[12] Edlinger S, Tenner E, Fruehwald-Pallamar J, Sprinzl G (2021). Magnetic resonance imaging and artefact reduction possibilities with the new active transcutaneous bone conduction implant (Bonebridge BCI602). Ann Otolaryngol Rhinol.

[13] Sprinzl G, Lenarz T, Ernst A, Hagen R, Wolf-Magele A, Mojallal H, Todt I, Mlynski R, Wolframm MD (2013). First European multicenter results with a new transcutaneous bone conduction hearing implant system: short-term safety and efficacy, (in eng). Otol Neurotol.

[14] Huber AM, Sim JH, Xie YZ, Chatzimichalis M, Ullrich O, Röösli C (2013). The Bonebridge: preclinical evaluation of a new transcutaneously-activated bone anchored hearing device, (in eng). Hear Res.

[15] Sprinzl GM, Schoerg P, Ploder M, Edlinger SH, Magele A (2021). Surgical experience and early audiological outcomes with new active transcutaneous bone conduction implant. Otol Neurotol.

[16] Gatehouse S, Noble W (2004). The speech, spatial and qualities of hearing scale (SSQ), (in eng). Int J Audiol.

[17] CoreTeam R (2013) R: A language and environment for statistical computing. R Foundation for Statistical Computing, Vienna, Austria. http://www.R-project.org/.

[18] Šikolová S, Hošnová D, Perceová K, Bartoš M, Kruntorád V, Urík M (2021). Retroauricular emphysema as a late complication after bonebridge implantation: case report. Ear Nose Throat J.

[19] Urik M, Hosnova D, Slapak I, Jancikova J, Odstrcilik J, Jarkovsky J, Baumgartner WD (2019). First experiences with a new adhesive bone conduction hearing device in children. Int J Pediatr Otorhinolaryngol.

[20] Der C, Bravo-Torres S, Pons N (2018). Active transcutaneous bone conduction implant: middle fossa placement technique in children with bilateral microtia and external auditory canal atresia. Otol Neurotol.

[21] Fan X, Wang Y, Wang P, Fan Y, Chen Y, Zhu Y, Chen X (2017). Aesthetic and hearing rehabilitation in patients with bilateral microtia-atresia. Int J Pediatr Otorhinolaryngol.

[22] Kulasegarah J, Burgess H, Neeff M, Brown CRS (2018). Comparing audiological outcomes between the Bonebridge and bone conduction hearing aid on a hard test band: Our experience in children with atresia and microtia. Int J Pediatr Otorhinolaryngol.

[23] Zernotti ME, Chiaraviglio MM, Mauricio SB, Tabernero PA, Zernotti M, Di Gregorio MF (2019). Audiological outcomes in patients with congenital aural atresia implanted with transcutaneous active bone conduction hearing implant. Int J Pediatr Otorhinolaryngol.

[24] Baumgartner WD, Hamzavi JS, Boheim K, Wolf-Magele A, Schlogel M, Riechelmann H, Zorowka P, Koci V, Keck T, Potzinger P, Sprinzl G (2016). A new transcutaneous bone conduction hearing implant: short-term safety and efficacy in children. Otol Neurotol.

[25] Ihler F, Volbers L, Blum J, Matthias C, Canis M (2014). Preliminary functional results and quality of life after implantation of a new bone conduction hearing device in patients with conductive and mixed hearing loss. Otol Neurotol.

[26] Seiwerth I, Frohlich L, Schilde S, Gotze G, Plontke SK, Rahne T (2021). Clinical and functional results after implantation of the bonebridge, a semi-implantable, active transcutaneous bone conduction device, in children and adults. Eur Arch Otorhinolaryngol.

[27] Weiss R, Leinung M, Baumann U, Weissgerber T, Rader T, Stover T (2017). Improvement of speech perception in quiet and in noise without decreasing localization abilities with the bone conduction device Bonebridge. Eur Arch Otorhinolaryngol.

[28] Vyskocil E, Liepins R, Kaider A, Blineder M, Hamzavi S (2017). Sound localization in patients with congenital unilateral conductive hearing loss with a transcutaneous bone conduction implant. Otol Neurotol.

[29] Vyskocil E, Riss D, Arnoldner C, Hamzavi JS, Liepins R, Kaider A, Honeder C, Fumicz J, Gstoettner W, Baumgartner WD (2017). Dura and sinus compression with a transcutaneous bone conduction device - hearing outcomes and safety in 38 patients. Clin Otolaryngol Off J ENT-UK Off J Netherlands Soc Oto Rhino Laryngol Cervico Fac Surg.

[30] Kraai T, Brown C, Neeff M, Fisher K (2011). Complications of bone-anchored hearing aids in pediatric patients, (in eng). Int J Pediatr Otorhinolaryngol.

[31] Zernotti ME, Sarasty AB (2015). Active bone conduction prosthesis: Bonebridge(TM), (in eng). Int Arch Otorhinolaryngol.

[32] Lassaletta L, Calvino M, Zernotti M, Gavilan J (2016). Postoperative pain in patients undergoing a transcutaneous active bone conduction implant (Bonebridge). Eur Arch Otorhinolaryngol.

[33] Hassepass F, Bulla S, Aschendorff A, Maier W, Traser L, Steinmetz C, Wesarg T, Arndt S (2015). The bonebridge as a transcutaneous bone conduction hearing system: preliminary surgical and audiological results in children and adolescents, (in eng). Eur Arch Otorhinolaryngol.

[34] Fan X, Yang T, Niu X, Wang Y, Fan Y, Chen X (2019). Long-term outcomes of bone conduction hearing implants in patients with bilateral microtia-atresia, (in eng). Otol Neurotol.

[35] Schmerber S, Deguine O, Marx M, Van de Heyning P, Sterkers O, Mosnier I, Garin P, Godey B, Vincent C, Venail F, Mondain M, Deveze A, Lavieille JP, Karkas A (2017). Safety and effectiveness of the Bonebridge transcutaneous active direct-drive bone-conduction hearing implant at 1-year device use. Eur Arch Otorhinolaryngol.

[36] Brkic FF, Riss D, Scheuba K, Arnoldner C, Gstöttner W, Baumgartner WD, Vyskocil E (2019). Medical, technical and audiological outcomes of hearing rehabilitation with the bonebridge transcutaneous bone-conduction implant: a single-center experience, (in eng). J Clin Med.

[37] Carnevale C, Til-Pérez G, Arancibia-Tagle DJ, Tomás-Barberán MD, Sarría-Echegaray PL (2019). Hearing outcomes of the active bone conduction system Bonebridge, (in eng|spa). Acta Otorrinolaringol Esp.

[38] Ngui LX, Tang IP (2018). Bonebridge transcutaneous bone conduction implant in children with congenital aural atresia: surgical and audiological outcomes, (in eng). J Laryngol Otol.

[39] Oh SJ, Goh EK, Choi SW, Lee S, Lee HM, Lee IW, Kong SK (2019). Audiologic, surgical and subjective outcomes of active transcutaneous bone conduction implant system (Bonebridge). Int J Audiol.

[40] Ratuszniak A, Skarzynski PH, Gos E, Skarzynski H (2019). The Bonebridge implant in older children and adolescents with mixed or conductive hearing loss: Audiological outcomes, (in eng). Int J Pediatr Otorhinolaryngol.

[41] Wang D, Ren R, Chen P, Yang J, Gao M, Liu Y, Zhao S (2020). Application of retrosigmoid sinus approach in Bonebridge implantation, (in eng). Acta Otolaryngol.

[42] Rohani SA, Bartling ML, Ladak HM, Agrawal SK (2020). The BONEBRIDGE active transcutaneous bone conduction implant: effects of location, lifts and screws on sound transmission. J Otolaryngol Head Neck Surg.

